# Colorectal cancer in patients with single versus double positive faecal immunochemical test results: A retrospective cohort study from a public tertiary hospital

**DOI:** 10.1371/journal.pone.0250460

**Published:** 2021-06-04

**Authors:** Tian Zhi Lim, Jerrald Lau, Gretel Jianlin Wong, Ker-Kan Tan

**Affiliations:** 1 Yong Loo Lin School of Medicine, National University of Singapore, Singapore, Singapore; 2 Saw Swee Hock School of Public Health, National University of Singapore, Singapore, Singapore; PLoS ONE, UNITED STATES

## Abstract

**Background:**

Screening for colorectal cancer (CRC) using the faecal immunochemical test (FIT) is widely advocated. Few studies have compared the rate of detecting colonoscopic pathologies in single compared to double FIT-positive follow-up colonoscopy-compliant individuals in a two-sample national FIT screening program.

**Objective:**

To compare CRC incidence in double FIT-positive versus single FIT-positive individuals using a retrospective cohort of patients from a tertiary hospital in Singapore.

**Design:**

Retrospective cohort study.

**Setting:**

Data was extracted from one public tertiary hospital in Singapore.

**Participants:**

1,422 FIT-positive individuals from the national FIT screening program who were referred to the hospital from 1st January 2017 to 31st March 2020 for follow-up consultation and diagnostic colonoscopy.

**Measurements:**

The exposure of interest was a positive result on both FIT kits. The main outcome was a follow-up diagnostic colonoscopy finding of CRC. The secondary outcome was a diagnostic colonoscopy finding of a colorectal polyp.

**Results:**

Incidence density of CRC was 1.15 and 13.10 per 100,000 person-months, in the single and double FIT-positive group, respectively. This resulted in an incidence rate ratio of 11.40 (95% CI = 4.34, 35.09). Colorectal polyp detection was significantly higher (p < 0.01) in the double (103 of 173 participants; 59.5%) compared to the single (279 of 671 participants; 41.6%) FIT-positive group.

**Limitations:**

The key limitation of this study was the relatively small cohort derived from a single tertiary hospital, as this had the effect of limiting the number of incident cases, resulting in comparatively imprecise CIs.

**Conclusions:**

Double FIT-positive individuals are significantly more likely to have a colonoscopy finding of incident CRC or premalignant polyp than single FIT-positive individuals. Clinicians and policymakers should consider updating their CRC screening protocols accordingly.

## Introduction

Colorectal cancer (CRC) screening has been shown to improve oncological outcomes by earlier detection and treatment of premalignant adenomatous polyps or CRC itself [1]. CRC screening modalities include the faecal immunochemical test (FIT), colonoscopy, flexible sigmoidoscopy, computed tomographic colonography and stool DNA testing. However, FIT remains the most cost-effective [1]. In the event that any FIT turns positive, a follow-up colonoscopy is recommended to complete the colonic evaluation [1, 2].

Singapore has adopted two-sample FIT in its national CRC screening program as this has been shown to increase sensitivity by potentially detecting neoplasms that may have been missed in one-sample FIT [3, 4]. The national CRC screening program invites age-eligible (e.g. more than age 50 years) average risk Singapore residents who are asymptomatic to complete a free two-sample FIT kit screening annually [3]. Average risk is defined as individuals with no family history of CRC and no personal history of adenomas, sessile serrated polyps, CRC or Inflammatory Bowel Disease (IBD) [5]. Residents are advised to collect two stool samples over two days before returning both the kits. The results of both kits are released together in the same sitting. Should either sample be positive, the individual would be contacted by a dedicated hospital coordinator to arrange for consultation and colonoscopy with a colorectal surgeon or gastroenterologist [6].

Individuals with two positive FIT samples (“double FIT-positive”)–which would have been sampled over two days–should intuitively be more likely to have colonic pathologies compared to individuals with only one positive FIT sample (“single FIT-positive”). However, few studies have examined these outcomes [4, 7]. Thus, this study aimed to compare CRC incidence in double FIT-positive versus single FIT-positive individuals using a retrospective cohort of patients from a tertiary hospital in Singapore.

## Methods

### Ethical approval

This study was ethically approved by the National Healthcare Group’s Domain Specific Review Board (NHG DSRB; Reference number: 2017/01260) in accordance with the Declaration of Helsinki. All data was analysed anonymously.

### Patient and public involvement statement

This research was done without patient involvement. Patients were not invited to comment on the study design and were not consulted to develop patient relevant outcomes or interpret the results. Patients were not invited to contribute to the writing or editing of this document for readability or accuracy. Informed consent was provided as part of enrolment to the national CRC screening program for both data analysis and publication.

### Study design and participants

The study population (N = 1,672) was a retrospective cohort enrolled through convenience sampling comprising all individuals who (I) had completed and submitted two FIT kits under the national CRC screening program, (II) received at least one positive FIT-result, and (III) were referred to a single tertiary institution (National University Hospital (NUH)) between 1^st^ January 2017 to 31^st^ March 2020 for follow-up consultation and diagnostic colonoscopy. The index date was the date of referral to the hospital. These individuals are referred directly to NUH by the program coordinator due to their residential address and not by personal choice. The socio-demographic distribution by residential address is representative of the national demographics.

Individuals were excluded from the study if they (I) had missing or incomplete results for either FIT kit, or (II) had transferred to another hospital at the point of data extraction (31^st^ December 2020). This resulted in a final sample of 1,422 participants, or 15.0% loss to follow up.

### Primary exposure and outcomes of interest

The exposure of interest was defined as a positive result on both FIT kits. The main outcome (incident CRC cases) was defined as a follow-up diagnostic colonoscopy finding of a CRC, regardless of staging. The secondary outcome was defined as a follow-up diagnostic colonoscopy finding of a colorectal polyp. Colonoscopy reports were verified by using the hospital’s electronic medical records system. Colonoscopy findings were grouped into normal, pre-malignant polyp and CRC. Pre-malignant polyp was defined as a tubular adenoma ≥ 10 mm in size, an adenoma of any size with villous features or high grade dysplasia, or a dysplastic serrated lesion of any size.

### Other participant factors

Apart from the main exposure and outcome of interest, our data included participants’ ethnicity (defined as Chinese, Malay, Indian, or Others), age, date of two-sample FIT kit collection, date of referral to NUH, and date of follow-up medical consultation and colonoscopy.

### Statistical analysis

For demographics, frequencies and proportions were used to report ethnicity, colonoscopy findings, predictive values and compliance to follow-up colonoscopy. Median and interquartile range (IQR) were used to summarise participant age. For colonoscopy-compliant participants, median and IQR was used to report follow-up time (in days) between date of referral to the hospital and date of colonoscopy. Incident CRC cases were summarised using frequencies and proportions.

To examine possible effects of confounding, chi-square tests were used to compare ethnicity and colonoscopy compliance, and Mann-Whitney U tests for age and follow-up time, between single and double FIT-positive groups.

A chi-square test was used to compare incident CRC cases and polyp detection between the groups. Incidence density (ID) of CRC cases was derived using person-months at risk, calculated from month of birth to the month of colonoscopy. Positive predictive value was tabulated as a proportion of positive colonoscopic findings over total colonoscopy performed in each group. Positive colonoscopic findings are defined as pre-malignant and cancer lesions. Incidence rate ratio (IRR) was used to compare relative risk of CRC between the single FIT-positive and double FIT-positive groups. Confidence intervals (95% CIs) were reported for IRR.

## Results

The median age of the sample was 66 years (IQR = 59–72 years). Overall compliance to follow-up colonoscopy was 51.7%. The distribution of sample demographics, colonoscopy compliance, and outcomes of interest between single and double FIT-positive patients can be found in Table 1.

**Table 1:** Distribution of age, ethnicity and follow-up colonoscopy compliance between single FIT-positive and double FIT-positive participants.

	Single FIT-positive	Double FIT-positive	*p*-value
	(*n* = 1345)	(*n* = 327)	
Median age, years (IQR)	65.1 (59.0–71.0)	68.0 (61.0–74.0)	< 0.01
Ethnicity, *n* (%)			0.08
Chinese	1253 (93.2)	298 (91.1)	
Malay	36 (2.7)	18 (5.5)	
Indian	36 (2.7)	6 (1.8)	
Other	20 (1.5)	5 (1.5)	
Colonoscopy compliance, *n* (%)	671 (49.9)	173 (52.9)	0.33
Follow-up time from referral to colonoscopy, median days (IQR)	53 (32–81)	49 (26–79)	0.12
Incident CRC cases, *n* (%)*	6 (0.9)	18 (10.4)	< 0.01
Colonoscopy with polyp finding, *n* (%)**	279 (41.6)	103 (59.5)	< 0.01

*Note: Denominator for proportions were based on colonoscopy-compliant participants only.

**Note: Denominator for proportions were based on colonoscopy-compliant participants, and excludes incident CRC cases detected.

Median follow-up time between date of referral to the hospital and date of colonoscopy was calculated for colonoscopy-compliant patients between the single (*n* = 670; median = 53 days, IQR = 32–81 days) and double FIT-positive (*n* = 172; median = 49 days, IQR = 26–79 days) groups; *U* = 53,222.5, *p* = 0.12.

The most common colonoscopic findings were pre-malignant polyps in both the single and double FIT-positive patients (see Table 2 for the breakdown of findings on follow-up colonoscopy). Any polyps detected were managed and removed in accordance to the discretion of the attending procedurist. The group of double FIT-positive patients had a higher positive predictive value (PPV) than those patients with single FIT-positive (69.9% versus 42.5%).

**Table 2:** Breakdown of findings on follow-up colonoscopy.

	Single FIT-positive	Double FIT-positive
	(*n* = 671) (%)	(*n* = 173) (%)
Colonoscopy findings		
Negative for lesions	348 (51.8)	46 (26.6)
Non-neoplastic polyps	38 (5.7)	6 (3.5)
Pre-malignant polyps	279 (41.6)	103 (59.5)
Colorectal cancer	6 (0.9)	18 (10.4)

Incidence density of CRC was 1.15 per 100,000 person-months in the single- and 13.10 per 100,000 person-months in the double FIT-positive group. This resulted in an incidence rate ratio of 11.40 (95% CI = 4.34, 35.09) (refer to Table 3).

**Table 3:** Incidence density of colorectal cancer, and incidence rate ratio, between single FIT-positive and double FIT-positive colonoscopy-compliant participants.

	Incident cases	Person-months at risk	ID per 100,000 person-months	IRR
				(95% CI)
Single FIT-positive (*n* = 671)	6	522402	1.15	
Double FIT-positive (*n* = 173)	18	137449	13.10	11.40
				(4.34, 35.09)

## Discussion

In colonoscopy-compliant patients, the double FIT-positive group was approximately 11 times more likely to have CRC and a significantly higher incidence of colorectal polyps than the single FIT-positive group. This correlated with the higher PPV in the group with double FIT-positive tests. These findings have significant implications to several stakeholders.

There is the need to expedite colonoscopy for those who are double FIT-positive to ensure prompt detection of CRC and premalignant polyps. With increasing advocacy on CRC screening and being current to screening recommendations, a higher number of patients who are at least single FIT-positive is expected if screening uptake increases. This could put strains on healthcare systems with finite facilities for colonoscopy, resulting in a need to prioritise which patients should more urgently undergo the procedure.

At the national level, countries that are still advocating CRC screening using only one-sample FIT should reconsider their approach. Double FIT-positive is likely to have increased specificity, resulting in fewer false positive findings during follow-up [4, 7]. Recent findings have also highlighted the significantly higher PPV of double FIT-positive compared to single FIT-positive in detecting CRC [4]. Although compliance rates of individuals performing two-sample FIT over two separate days are likely to be slightly lower than one-sample FIT, this can be managed through public education and targeted interventions [7]. Countries concerned about costs to the health system should consider performing a cost-effectiveness analysis, which will likely favour a two-sample FIT program [7].

More work is also required to reinforce the importance of prompt follow-up after a positive FIT result. As observed in our study and the literature, colonoscopy compliance rate after a positive FIT is approximately 50% [8]. Using simple arithmetic, another 33 CRC cases could have been diagnosed and managed promptly from our cohort should there have been perfect compliance. Prior literature has suggested a combination of patient and provider factors influencing follow-up compliance [8]. As these have been known to vary between populations, public health professionals and policymakers must understand the target population-specific barriers and facilitators in order to develop tailored, cost-effective interventions to improve compliance to follow-up colonoscopy. This can be achieved through a process evaluation to pin-point specific interventions from referral at the national screening level, to the arrival at the clinic for the medical consultation and eventually having undergone the colonoscopic evaluation.

The key strengths of this study were (I) the representativeness of the sample to the average-risk study population recommended to our hospital via the national screening programme, with low loss to follow up (15.0%), and (II) the ability to verify outcomes of interest via the hospital’s clinical database. The key limitation of this study was the relatively small cohort derived from referrals of the national CRC screening program to a single tertiary hospital, as this had the effect of limiting the number of incident cases, resulting in comparatively imprecise CIs. The various ethnic groups in Singapore could also be a potential confounder given possible difference in knowledge, beliefs and attitude towards CRC screening. Nonetheless, our findings observed similar socio-demographic characteristics between both groups. This should be the case and suggests that our data is representative of national demographics as FIT-positive hospital referrals are performed geographically on a national level, and population sociodemographic characteristics are similarly distributed across Singapore [9]. Although there is certainly the need to validate our findings in larger two-sample FIT cohorts, the significant difference in the rates of CRC detected should prompt clinicians and policymakers to consider examining this issue with greater urgency.

## Supporting information

S1 File (XLSX)
